# Prolonged *ex-situ* heart perfusion preserves cardiac output following orthotopic transplantation in a porcine model

**DOI:** 10.3389/fcvm.2026.1805585

**Published:** 2026-04-15

**Authors:** Zhuldyz Nurmykhametova, Rymbay Kaliyev, Timur Lesbekov, Linar Faizov, Assel Medressova, Aiym Turarova, Yerik Zuparov, Yuriy Pya

**Affiliations:** 1 Department of Perfusiology and Assisted Circulation Laboratory, University Medical Center Corporate Fund, Astana, Kazakhstan; 2 Department of Adult Cardiac Surgery, University Medical Center Corporate Fund, Astana, Kazakhstan; 3 Department of Anesthesiology, University Medical Center Corporate Fund, Astana, Kazakhstan.

**Keywords:** ECMO, *ex-situ* heart perfusion, heart transplantation, normothermic perfusion, prolonged preservation

## Abstract

The safe ischemic window for static cold storage in heart transplantation is around 4–6 h, limiting donor retrieval distance and contributing to organ discard. We aim to assess whether a porcine heart preserved for 24 h using a novel ECMO-based ESHP system can maintain function after orthotopic transplantation. Five-orthotopic allogeneic heart transplantation were preserved for 24 h on a custom ECMO-based ESHP circuit at 37 °C, with coronary perfusion pressure 60–70 mmHg, and metabolic/hormonal supplementation. Hemodynamic, metabolic, and oxygenation parameters were measured using pulmonary arterial thermodilution via a Swan-Ganz catheter. Recipients were monitored for 4 h post-transplantation: cardiac output increased from 5.94 ± 0.16 L/min (baseline) to 6.78 ± 0.12 L/min (immediately post-transplant) (*p* = 0.006) and 7.06 ± 0.16 L/min at 4 h following transplantation (*p* = 0.017). Despite significant reductions in hemoglobin and arterial oxygen content, oxygen delivery remained adequate, oxygen consumption increased significantly at 4 h following transplantation to 172.88 ± 7.60 mL/min/m² (*p* = 0.0322 vs. donor) and lactate levels stayed low. No evidence of acute graft dysfunction was observed. Normothermic preservation of the heart with our institutional ESHP protocol for 24 h maintained graft viability and function in a porcine transplantation model.

## Introduction

Heart transplantation remains limited due to the shortage of suitable donor organs and by preservation constraints. Static cold storage allows only 4–6 h of safe ischemic time, after which the risk of primary graft dysfunction (PGD) and early mortality increases sharply (1). This short preservation window restricts retrieval distance and leads to the discard of otherwise viable grafts.

Normothermic *ex-situ* heart perfusion (ESHP) offers an alternative by maintaining the heart in a warm, oxygenated, and metabolically active state (2). This approach may be particularly valuable in enabling functional assessment and repair of marginal grafts, including those from donation after circulatory death (DCD). These advantages offer the potential to expand the donor pool and improve organ utilization. However, prolonged ESHP has been shown to be associated with myocardial functional decline due to metabolic disturbances, oxidative stress, and inflammatory activation (3, 4). Recent studies suggest that optimized perfusion protocols can attenuate these effects, preserving myocardial contractility and reducing edema during extended perfusion time (5, 6). Yet, it remains unknown whether a donor heart preserved for 24 h ESHP can recover and sustain adequate hemodynamic and metabolic performance after transplantation. To address this question, we evaluated post-transplant functional recovery of five hearts after 24 h normothermic preservation using a novel ECMO-based ESHP platform in our institution. The aim of our study was to evaluate whether a porcine heart preserved for 24 h using a novel ESHP system and protocol can maintain stable central hemodynamics and adequate tissue oxygenation following orthotopic transplantation.

## Materials and methods

### Animal model

This study was approved by the National Research Cardiac Surgery Center's Animal Care and Use Committee (Protocol No. 2022/01-121). Five orthotopic allogeneic heart transplants were performed in domestic pigs. Five orthotopic allogeneic heart transplantations were performed in domestic pigs. Both donor and recipient animals weighed 90–95 kg and were 6–8 months of age. All donor pigs were hemodynamically stable prior to procurement and did not require inotropic or vasopressor support.

### Anesthesia and monitoring

Pigs fasted for 12 h preoperatively. Premedication was performed with xylazine (1–2 mg/kg, SC) administered 15–20 min before transfer to the operating room. After auricular vein catheterization, anesthesia was induced with intravenous propofol (2 mg/kg/h), followed by endotracheal intubation. Animals were mechanically ventilated (volume-control mode, tidal volume 8–9 mL/kg, PEEP 7–10 cmH₂O, FiO₂ 35%), with respiratory rate adjusted to maintain end-tidal CO₂ at 35–40 mmHg. Anesthesia was maintained with fentanyl (2 μg/kg/h) and sevoflurane (MAC 0.5%–1.5%).

The right carotid artery was catheterized for invasive arterial pressure monitoring and arterial blood gas analysis. A triple-lumen catheter was inserted in the right internal jugular vein for central venous pressure monitoring and drug administration. A Swan-Ganz thermodilution catheter (7.5F, Edwards Lifesciences, USA) was advanced to the pulmonary artery via an 8.5F introducer for central hemodynamic measurements and mixed venous blood sampling. Heart rate, ECG, invasive arterial pressure, central venous pressure, pulmonary artery pressure, oxygen saturation, end-tidal CO₂, MAC, and esophageal temperature were continuously recorded.

### Donor surgical procedure

A median sternotomy was performed, and the pericardium was opened. Unfractionated heparin (400 IU/kg) was administered intravenously for systemic anticoagulation. A single-stage venous cannula was inserted into the right atrium for blood collection, and exsanguination was used to prime the ESHP circuit. A cardioplegia cannula was placed in the ascending aorta.

The aorta was cross-clamped, and 1 L of warm blood based cardioplegia was administered. The IVC and left atrial appendage were incised to decompress the heart. The heart was excised after division of the great vessels. For machine perfusion, the aorta was trimmed below the cardioplegia cannula suture to prevent leakage and connected to the aortic connector and secured. The pulmonary artery was cannulated and tied using a 4–0 Prolene purse-string suture. All left atrial veins were closed leaving a left ventricular vent for drainage. The IVC and SVC were ligated. The aortic site was connected to the aortic port of the heart box, and epicardial pacing wires were placed for optional pacing during perfusion. *Ex-situ* reperfusion was initiated approximately 25 min after aortic cross-clamping.

### *Ex-situ* heart preservation

**Figure 1:**
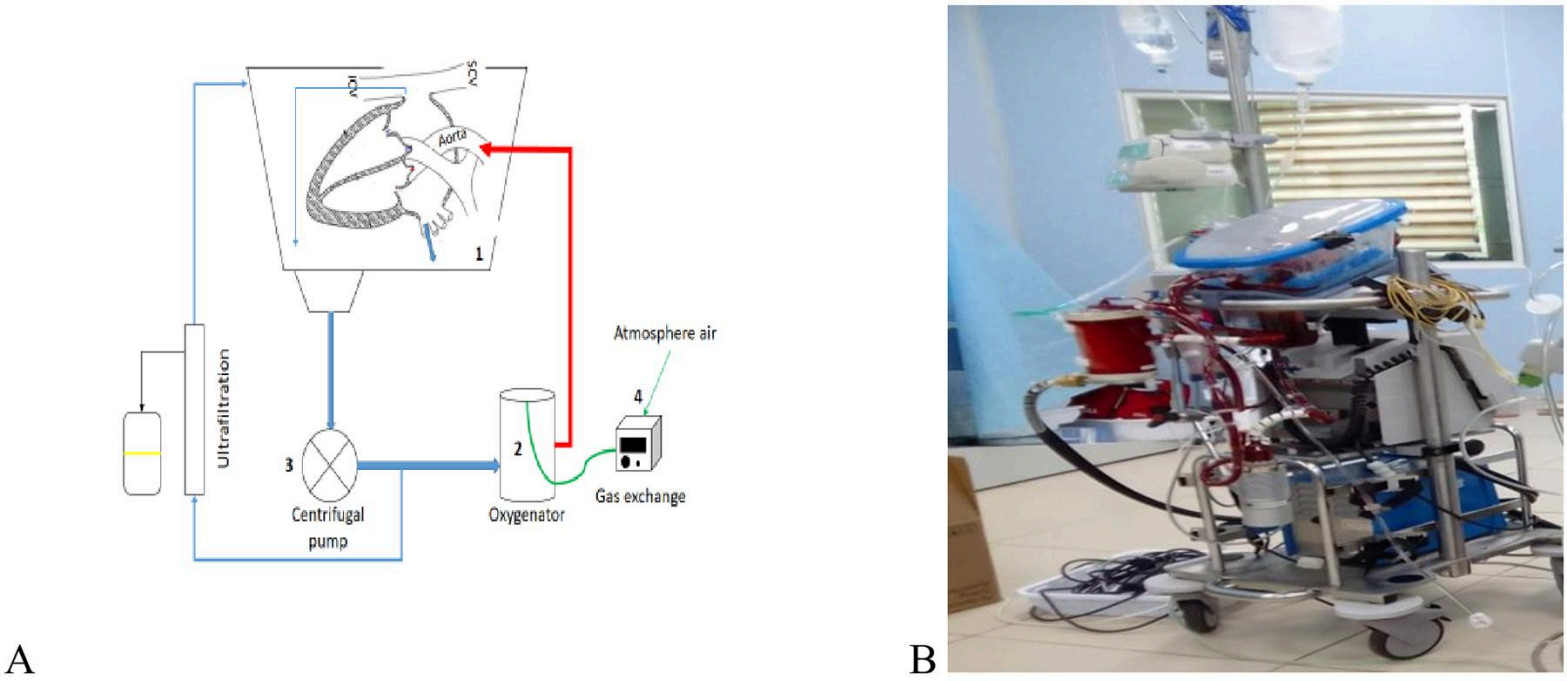
Hemodynamic and hematological changes following orthotopic heart transplantation after 24 h *ex-situ* heart perfusion. The figure compares cardiac output **(A)**, cardiac index **(B)**, systemic vascular resistance index **(C)**, and hemoglobin **(D)** in donor (1), recipient after weaning from cardiopulmonary bypass (2), and 4 h post-transplant (3). Data are presented as mean ± SEM, *n* = 5. Statistical analysis: paired *t*-test; *p* < 0.05, *p < 0.01, **p < 0.0001.

All hearts were maintained on ESHP for 24 h. The perfusion circuit (Figure 1) was based on the ECMO platform manufactured by Medos AG (Stolberg, Germany). A centrifugal pump was operated in flow-adjustment mode, with rpm titrated to maintain a mean aortic pressure of 60–70 mmHg, resulting in coronary flows of 500–800 mL/min. Aortic root pressure was continuously monitored via a calibrated pressure transducer connected to the aortic cannula.

Gas exchange was provided by a compatible polymethylpentene hollow-fiber oxygenator (Medos AG). The circuit consisted of 3/8-inch (9.5 mm ID) ECMO tubing with standard 3/8-inch connectors and luer-lock interfaces for monitoring lines. Coronary sinus effluent was collected via gravity drainage into a custom-designed, closed integrated reservoir developed in our institution.

An inline hemoconcentrator (ultrafiltration module; Medos AG) was incorporated into the circuit and operated in continuous mode. Ultrafiltration (UF) rate was adjusted according to perfusate volume and concentration targets, with continuous monitoring of transmembrane pressure in accordance with manufacturer specifications.

UF rates were titrated between 10 and 100 mL/h depending on real-time assessment of reservoir volume, hematocrit trends, and perfusate electrolyte concentrations. Ultrafiltration was increased in the presence of volume overload or hemodilution and reduced or temporarily discontinued if hematocrit approached the lower target limit.

The perfusate consisted of autologous whole blood diluted with balanced crystalloid solution (Plasma-Lyte A) at an approximate ratio of 1:1 to achieve a target hematocrit of 20%–25%. Electrolyte composition was maintained within physiologic range [Na+ 135–145 mmol/L, K+ 4.0–5.0 mmol/L, Ca²+ 1.0–1.2 mmol/L (ionized), Mg²+ 0.8–1.2 mmol/L]. Glucose was supplemented as required to maintain normoglycemia. Additional additives included sodium bicarbonate for acid–base correction and insulin as needed. No albumin or additional colloids were added to the perfusate.

Hemoglobin concentration and glucose levels were measured every 2 h during ESHP to monitor metabolic stability and oxygen-carrying capacity. Acid–base balance was continuously monitored, and perfusate pH was maintained at 7.4 (±0.1 SD) through adjustment of gas flow to the oxygenator and administration of sodium bicarbonate supplementation as required.

### Recipient procedure

Following the end of ESHP, the heart remained ischemic during transport and implantation for ± 40 min until release of the aortic cross-clamp in the recipient, resulting in a total preservation time from donor cross-clamp to recipient reperfusion of 25 h. Preparation, anesthesia, and monitoring were identical to those used for donor pigs. Each recipient animal underwent a median sternotomy, followed by systemic heparinization. Cardiopulmonary bypass (CPB) was established via aortic and bicaval cannulation. CPB was performed using non-pulsatile flow of 2.4–2.6 L/min/m², with temperature maintained at 34–35 °C, under standard physiologic monitoring. The native heart was arrested and excised, leaving appropriate cuffs of the great vessels and left atrium for graft implantation. The donor heart aorta was cross-clamped, and 1 L of warm blood based cardioplegia was administered. The preserved donor heart was transplanted orthotopically in the following sequence: left atrium, pulmonary artery, ascending aorta, IVC, and SVC. After completion of all anastomoses and thorough de-airing, the aortic cross-clamp was removed to restore coronary perfusion. The cardioplegia administration was repeated every 20 min. Once satisfactory cardiac function and stable hemodynamics were confirmed, the animal was weaned from CPB. The sternotomy was closed in standard fashion with placement of thoracic drains, and the recipient was monitored for 4 h postoperatively to evaluate graft performance.

#### Outcomes measured

##### Hemodynamic parameters

Central hemodynamic and metabolic parameters were assessed at three time points: (1) baseline, in donor animals after anesthesia induction and stabilization; (2) in recipient pigs after weaning from CPB; and (3) four hours after sternal closure during the postoperative period. Hemodynamic measurements were obtained using the pulmonary arterial thermodilution technique via a Swan-Ganz catheter. At each time point, three injections of 10 mL cold saline were performed, and the average value was recorded. All parameters were indexed to body surface area (BSA) and calculated as:

$$\mathrm{BSA}(m^{2})=0.0734\times (\mathrm{body}\;\;\mathrm{weight}\;\mathrm{in}\;\mathrm{kg}{)}^{0.656\;}$$

The variables measured, along with their formulas and units, are summarized in Table 1.

**Table 1:** Measured hemodynamic, metabolic and oxygenation parameters.

Parameter	Abbreviation	Formula	Units
Hemodynamic Parameters			
Cardiac Output	CO	Measured by thermodilution	L/min
Cardiac Index	CI	CO/BSA	L/min/m²
Systemic Vascular Resistance	SVR	[(MAP—CVP) × 80]/CO	dyn·s·cm−⁵
Systemic Vascular Resistance Index	SVRI	[(MAP—CVP) × 80]/CI	dyn·s·cm−⁵·m²
Pulmonary Vascular Resistance	PVR	[(mean PAP—PCWP) × 80]/CO	dyn·s·cm−⁵
Pulmonary Vascular Resistance Index	PVRI	[(mean PAP—PCWP) × 80]/CI	dyn·s·cm−⁵·m²
Stroke Volume	SV	(CO × 1,000)/HR	mL
Stroke Index	SI	SV/BSA	mL/m²
Systemic Oxygen Utilization Index	SIRV	0.0136 × (MAP—CVP) × SI	mmHg·mL/m²
Pulmonary Oxygen Utilization Index	SILV	0.0136 × (MAP—PCWP) × SI	mmHg·mL/m²
Metabolic and Oxygenation Parameters			
Arterial Oxygen Content	CaO₂	(0.0138 × Hb × SaO₂) + (0.0031 × PaO₂)	mL/dL
Venous Oxygen Content	CvO₂	(0.0138 × Hb × SvO₂) + (0.0031 × PvO₂)	mL/dL
Oxygen Delivery	DO₂	CI × CaO₂	mL/min/m²
Oxygen Consumption	VO₂	CI × (CaO₂—CvO₂)	mL/min/m²
Oxygen Extraction Ratio	KYO₂	(VO₂/DO₂) × 100	%
Serum Lactate		Measured enzymatically from arterial blood	mmol/L

##### Statistical analysis

Statistical analyses were performed using GraphPad Prism version 10.1.2 (GraphPad Software, San Diego, CA, USA). Data are expressed as mean ± standard error of the mean (SEM). Comparisons between paired measurements were performed using paired t-tests, with results cross-checked using the non-parametric Wilcoxon signed-rank test to confirm robustness. Both methods yielded consistent significance patterns; for brevity, only t-test results are reported in the tables. *P*-values < 0.05 were considered statistically significant.

## Results

Orthotopic heart transplantation was successfully completed in all five recipient animals following 24 h ESHP. Donor pigs demonstrated stable hemodynamics prior to organ retrieval without inotropic or vasopressor support. No intraoperative mortality occurred, and all recipients achieved hemodynamic stability with only mild pharmacological support [norepinephrine (0.05–0.15 mcg/kg/min) was administered in three recipient pigs]. Hemodynamic and metabolic changes before and after orthotopic heart transplantation following 24 h ESHP are presented in Table 2.

**Table 2:** Hemodynamic and metabolic changes before and after orthotopic heart transplantation following 24 h ESHP (*n* = 5).

Parameter	Donor	Post-CPB	4 h post-transplant	*p* (donor vs. post-CPB)	*p* (donor vs. 4 h post-transplant)
Contractile function					
CO (L/min)	5.94 ± 0.16	6.78 ± 0.12	7.06 ± 0.16	**0**.**006**	**0**.**017**
CI (L/min/m²)	3.96 ± 0.13	4.56 ± 0.19	4.74 ± 0.20	**0**.**023**	**0**.**022**
SV (mL)	68.16 ± 3.61	64.38 ± 2.47	73.22 ± 3.29	0.114	0.130
SI (mL/m²)	45.36 ± 1.82	43.06 ± 2.10	48.98 ± 2.49	0.294	0.217
SILV (mmHg·mL/m²)	42.86 ± 2.69	42.48 ± 3.69	46.90 ± 2.93	0.912	**0**.**031**
SIRV (mmHg·mL/m²)	10.52 ± 1.18	9.72 ± 0.91	10.74 ± 1.02	0.495	0.823
HR (bpm)	87.80 ± 3.48	105.80 ± 3.44	97.00 ± 3.94	**<0**.**001**	**0**.**011**
Systemic Hemodynamics					
MAP (mmHg)	81.20 ± 2.24	82.20 ± 3.23	81.20 ± 1.50	0.789	1.000
CVP (mmHg)	6.40 ± 0.40	5.40 ± 0.98	7.00 ± 0.45	0.266	0.426
SVR (dyn·s·cm−⁵)	1,008.78 ± 28.72	906.04 ± 20.06	843.26 ± 30.63	0.088	0.040
SVRI (dyn·s·cm−⁵·m²)	1,513.70 ± 58.52	1,359.62 ± 53.73	1,268.62 ± 76.78	0.142	0.069
Pulmonary Hemodynamics					
Mean PAP (mmHg)	23.60 ± 1.35	21.80 ± 1.07	23.00 ± 0.84	0.266	0.763
PCWP (mmHg)	12.00 ± 0.55	10.00 ± 0.84	10.80 ± 0.86	0.034	0.359
PVR (dyn·s·cm−⁵)	156.76 ± 15.36	137.76 ± 12.28	138.82 ± 14.89	0.320	0.497
PVRI (dyn·s·cm−⁵·m²)	233.22 ± 23.71	197.68 ± 17.53	202.86 ± 21.96	0.238	0.508
Metabolic and Oxygenation Parameters					
Hb (g/L)	132.40 ± 1.02	99.00 ± 1.58	100.20 ± 1.07	<0.001	<0.001
CaO₂ (mL O₂/L)	181.34 ± 1.77	135.98 ± 1.89	137.96 ± 1.51	<0.001	<0.001
CvO₂ (mL O₂/L)	132.70 ± 3.55	98.06 ± 2.38	101.06 ± 0.66	<0.001	<0.001
DO₂ (mL/min/m²)	718.76 ± 16.71	622.06 ± 17.16	650.66 ± 17.05	0.008	0.098
VO₂ (mL/min/m²)	171.66 ± 12.08	181.06 ± 8.59	172.88 ± 7.60	0.531	0.929
KYO₂ (%)	26.80 ± 1.85	29.92 ± 1.53	27.20 ± 0.92	0.319	0.879
Lactate (mmol/L)	1.18 ± 0.14	2.28 ± 0.38	1.66 ± 0.18	0.055	0.085

Bold values indicate statistically significant differences (*p* < 0.05).

### Contractile function

**Figure 2:**
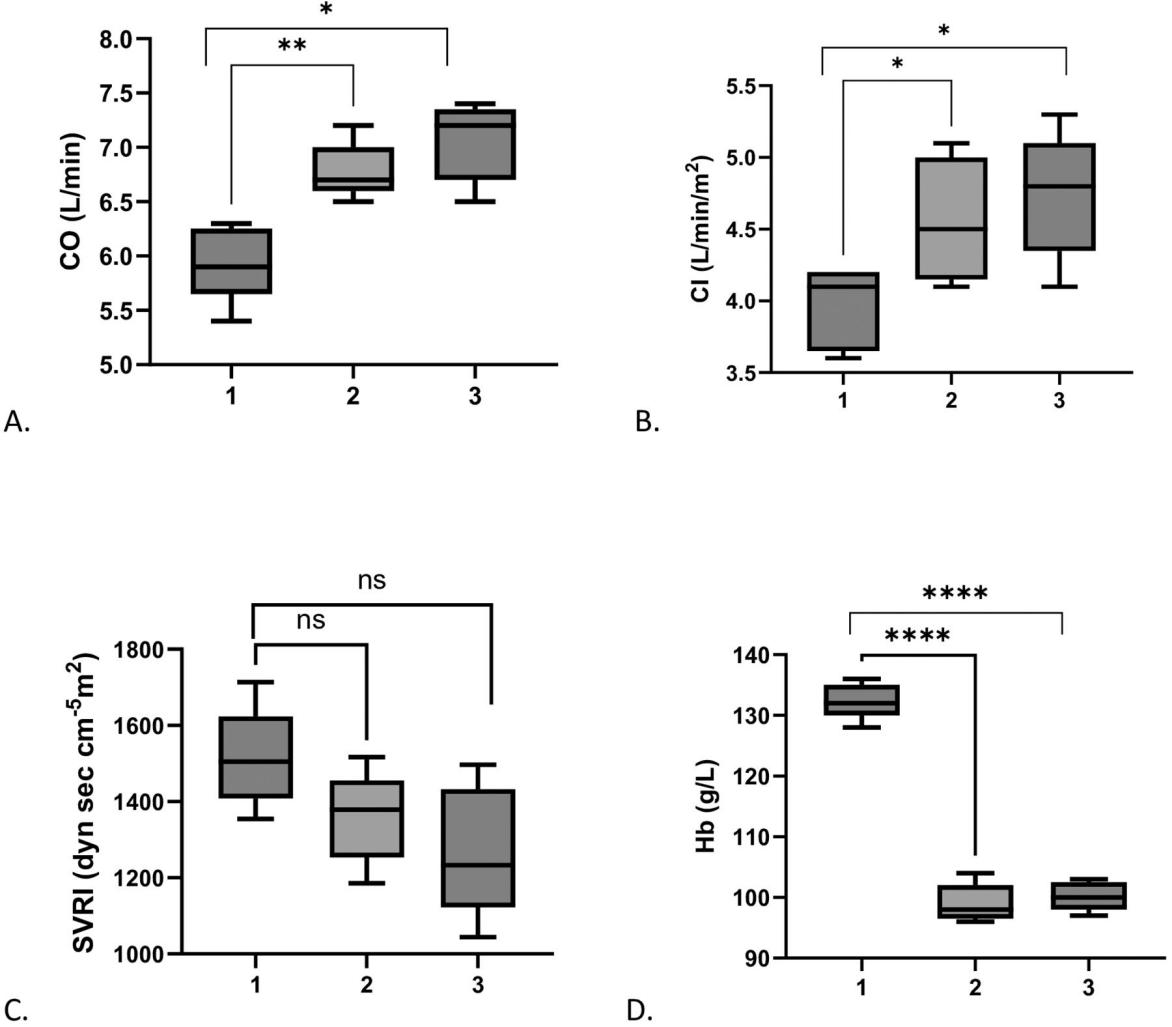
Custom ECMO-based *ex situ* heart perfusion (ESHP) system. **(A)** Schematic of the mobile ECMO-based circuit used for prolonged *ex situ* heart preservation, including a centrifugal pump, oxygenator, heat exchanger, reservoir, and ultrafiltration unit connected through a closed perfusion loop. **(B)** Photograph of the assembled mobile ESHP circuit used in the experimental setup.

Myocardial contractile function was preserved and showed early improvement following transplantation. **Cardiac output** (CO, Figure 2A) increased from a donor baseline of **5.94** **±** **0.16 L/min** to 6.78 ± 0.12 L/min immediately post-transplant (+14%, *p* = 0.006), and further to 7.06 ± 0.16 L/min at 4 h post-sternal closure (+19% from baseline, *p* = 0.017). **Cardiac index** (CI, Figure 2B) rose from 3.96 ± 0.13 L/min/m² to 4.56 ± 0.19 (*p* = 0.023) and 4.74 ± 0.20 (*p* = 0.022). **Heart rate (HR)** increased significantly post-transplant (+21%, *p* < 0.001) before decreasing slightly by 4 h (*p* = 0.011 vs. donor). The elevated HR likely reduced diastolic filling time, contributing to a transient fall in **stroke volume (SV)** and **stroke index (SI)** after weaning from cardiopulmonary bypass. Both parameters showed numerical recovery by 4 h (+7% vs. baseline), though changes were not statistically significant. The **stroke index of the left ventricle (SILV)** showed significant increase at 4 h following transplantation (from 42.86 ± 2.69 mL/m² to 46.90 ± 2.93 mL/m², *p* = 0.0309).

### Systemic hemodynamics

Mean arterial pressure (MAP) remained stable across all time points (donor: 81.20 ± 2.24 mmHg; 4 h: 81.20 ± 1.50 mmHg, *p* = 1.000). Central venous pressure (CVP) varied slightly but stayed within physiological limits (*p* > 0.05). Systemic vascular resistance (SVR) fell from 1,008.78 ± 28.72 dyn·s/cm⁵ to 906.04 ± 20.06 post-transplant (*p* = 0.088) and 843.26 ± 30.63 at 4 h (*p* = 0.040). Systemic vascular resistance index (SVRI, Figure 2C) showed a similar declining trend, though not statistically significant.

### Pulmonary hemodynamics

Mean pulmonary artery pressure (PAP) remained stable within physiological range. Pulmonary capillary wedge pressure (PCWP) decreased immediately post-transplant (12.00 ± 0.55 mmHg to 10.00 ± 0.84 mmHg, *p* = 0.0341) before partial recovery by 4 h (*p* = 0.359). Pulmonary vascular resistance (PVR) and pulmonary vascular resistance index (PVRI) showed no significant variation across time points. No signs of acute right ventricular dysfunction or pulmonary hypertension post-transplant were seen.

### Metabolic and oxygenation parameters

Hemoglobin (Hb, Figure 2D) decreased significantly following cardiopulmonary bypass, falling from 132.40 ± 1.02 g/L in donors to 99.00 ± 1.58 g/L post-transplant (−25%, *p* < 0.001) and remaining low at 100.20 ± 1.07 g/L at 4 h (*p* < 0.001 vs. donor). This reduction was paralleled by a significant decline in arterial oxygen content (CaO₂) from 181.34 ± 1.77 mL O₂/L at baseline to 135.98 ± 1.89 mL O₂/L post-transplant and 137.96 ± 1.51 mL O₂/L at 4 h (both *p* < 0.001). Venous oxygen content (CvO₂) demonstrated a similar pattern, decreasing from 132.70 ± 3.55 mL O₂/L to 98.06 ± 2.38 mL O₂/L post-transplant and 101.06 ± 0.66 mL O₂/L at 4 h (*p* < 0.001 for both comparisons).

Oxygen delivery (DO₂) declined significantly from 718.76 ± 16.71 mL/min/m² in donors to 622.06 ± 17.16 mL/min/m² post-transplant (*p* = 0.008), with partial recovery to 650.66 ± 17.05 mL/min/m² at 4 h (*p* = 0.098). Oxygen consumption (VO₂) remained unchanged immediately post-transplant (171.66 ± 12.08 vs. 181.06 ± 8.59 mL/min/m², *p* = 0.531) but increased significantly at 4 h following transplantation to 172.88 ± 7.60 mL/min/m² (*p* = 0.0322 vs. donor). Mixed venous oxygen saturation (SvO₂), venous PO₂ (PvO₂), and oxygen extraction ratio (KYO₂) remained stable across all timepoints (*p* > 0.05). Lactate levels rose slightly after transplantation (1.18 ± 0.14–2.28 ± 0.38 mmol/L) but did not reach statistical significance (*p* = 0.055 post-transplant; *p* = 0.085 at 4 h). Despite hemodilution-induced reductions in Hb and oxygen content, oxygen delivery remained adequate and oxygen consumption increased during early reperfusion, with stable SvO₂ and lactate suggesting preserved global oxygen balance.

## Discussion

The post-transplant increase of cardiac output and cardiac index, coupled with stable mean arterial and pulmonary pressures, underscores preserved ventricular contractility and favorable vascular adaptation after prolonged preservation of the donor hearts. The significant rise in heart rate observed upon weaning from cardiopulmonary bypass was likely multifactorial, reflecting hemodilution, perioperative blood loss, and stress-related sympathetic activation. Concurrent decreases in systemic vascular resistance might be influenced by reperfusion-related inflammatory mediators and residual anesthetic effects (7, 8). Importantly, these hemodynamic shifts occurred without evidence of pump failure.

Oxygen transport variables further support the conclusion of functional graft viability. While Hb concentration and arterial oxygen content declined significantly after transplantation, global oxygen delivery remained within physiologically acceptable limits. Mixed venous oxygen saturation and oxygen extraction ratio remained stable, indicating that tissue oxygenation was not compromised. The significant increase in oxygen consumption by 4 h post-implantation likely reflects rising myocardial metabolic activity and cellular recovery following reperfusion. The absence of significant lactate accumulation, combined with stable venous oxygen content, suggests sustained aerobic metabolism and an adequate match between oxygen supply and demand throughout the observation period.

This initial results sugests substantial extension of the conventional preservation window. The only FDA-approved heart perfusion system (TransMedics Organ Care System) typically allows for 6–8 h of preservation of the donor heart before transplantation. By successfully bridging a full 24 h interval between heart procurement and transplantation, our study points to the possibility of expanding the donor pool. This could enable logistical flexibility without compromising graft viability.

Our results align with emerging evidence from preclinical models that extended-duration perfusion is feasible with careful protocol optimizations. For example, Spencer *et al.* recently reported that adult pig hearts could be perfused *ex-situ* for 24 h at normothermia with preserved function throughout (9). Likewise, Johnson et al. demonstrated that porcine hearts subjected to 24 h of cold static storage followed by 5 h of normothermic perfusion were still transplantable, with recipients being successfully weaned off cardiopulmonary support (10). These studies support the concept that the viability of donor hearts can be maintained far beyond the historical 6 h cutoff when adequate oxygenation, nutrients, and waste removal are provided. Our work builds on this progress by confirming that a 24 h perfused heart not only remains metabolically active but can also integrate and function in an orthotopic transplant setting.

It is important to note that prolonged warm perfusion is known to impose physiological stresses on the graft. Normothermic *ex situ* perfusion can trigger a sustained inflammatory response, oxidative stress, and other ischemia–reperfusion injury mechanisms that contribute to a gradual decline in cardiac functional performance over time. Indeed, without targeted interventions, many perfused hearts show deteriorating contractility and accumulating metabolic derangements as perfusion is extended. In our protocol, several strategies were employed to mitigate these issues, including controlled coronary flow to prevent pressure overload, hormonal and metabolic supplementation (insulin–glucose, bicarbonate, etc.) and continuous hemofiltration to remove inflammatory mediators and toxins. The effectiveness of these measures are reflected in the stable cardiac performance observed. Despite 24 h on the perfusion circuit, the transplanted hearts did not demonstrate signs of progressive failure: lactate levels remained low, and cardiac output indices showed an upward trend during early reperfusion. Notably, gross examination of the hearts after 24 h perfusion did not demonstrate visible myocardial edema. Ventricular wall thickness and tissue turgor appeared subjectively comparable to the pre-perfusion state. However, as formal histologic assessment was not performed, the presence of microscopic interstitial edema cannot be definitively excluded.

Extending viable preservation times to a full day opens the door to remote organ retrieval on a global scale. Moreover, it creates opportunities to *assess* and *rehabilitate* donor hearts *ex-situ*, by administering cardioprotective therapies, performing genetic or regenerative interventions. These advantages are unattainable with static cold storage.

Despite these encouraging findings, several important limitations must be acknowledged. The small sample size (*n* = 5) and short postoperative observation window (4 h) inherently limit the generalizability of the results. These data should therefore be interpreted as preliminary proof of concept rather than definitive evidence that 24 h normothermic perfusion ensures graft viability. Additionally, the absence of a concurrent control arm precludes firm attribution of the observed outcomes. Future studies should include larger experimental cohorts, appropriate comparator groups, and longer postoperative follow-up to clarify the reproducibility, durability, and mechanistic implications of prolonged normothermic heart preservation.

## Data Availability

The raw data supporting the conclusions of this article will be made available by the authors, without undue reservation.
